# Transcription of Nitrogen Fixation Genes Is Enhanced at Unfavorably High Oxygen Concentrations for Diazotrophic Growth in a Methane-oxidizing Bacterium

**DOI:** 10.1264/jsme2.ME25032

**Published:** 2025-11-06

**Authors:** Argen Adem Abdela, Rina Shinjo, Takeshi Watanabe, Susumu Asakawa, Sachiko Masuda, Arisa Shibata, Ken Shirasu, Kiwamu Minamisawa, Shusei Sato, Hisayuki Mitsui

**Affiliations:** 1 Graduate School of Life Sciences, Tohoku University, 2–1–1 Katahira, Aoba-Ku, Sendai, 980–8577, Japan; 2 Graduate School of Bioagricultural Sciences, Nagoya University, Furo-cho, Chikusa, Nagoya 464–8601, Japan; 3 RIKEN Center for Sustainable Resource Science, RIKEN-TRIP, 1–7–22 Suehiro-cho, Tsurumi, Yokohama 230–0045, Japan

**Keywords:** methane, nitrogen fixation, methanotroph, nitrogenase, transcription

## Abstract

Since nitrogenase is intrinsically sensitive to oxygen (O_2_), diverse aerobic diazotrophs need strategies to cope with nitrogenase damage by O_2_. In the present study, we investigated the mechanisms by which aerobic methane-oxidizing bacteria (methanotrophs) enable the concurrent activities of methane monooxygenase, which uses O_2_, and nitrogenase in the cytoplasm of the same cell. By using ^15^N labeling, we confirmed the capacity of alphaproteobacterial methanotroph *Methylosinus* sp. 3S-1 for nitrogen fixation and diazotrophic growth across a wide range of O_2_ concentrations <20%. When the initial O_2_ concentration was increased from 2 to 20% in a diazotrophic culture, similar decreases were observed in fixed nitrogen and NifH protein levels. In contrast, the mRNA levels of nitrogen fixation genes (*nif* genes) markedly increased and remained elevated for the duration of slow growth at high O_2_ concentrations. This pattern of *nif* expression in response to O_2_ may be attributed to the properties of the *nif*-specific transcriptional regulator NifA. The present results suggest that the increase in *nif* transcription is one of the strategies by which this methanotroph maintains nitrogen fixation on the background of aerobic methane oxidation.

Biological nitrogen fixation, facilitated by the complex metalloenzyme nitrogenase, is a process by which inert dinitrogen gas (N_2_) is metabolically converted into the tractable nitrogen form, ammonia, under physiological conditions. This enzyme consists of two components, the iron protein (NifH) and molybdenum–iron protein (NifDK): the former contains a simple iron-sulfur cluster [4Fe–4S] and functions to donate electrons to the latter component, while the latter contains two complex metalloclusters, the P cluster [8Fe–7S] and the Fe-Mo cofactor [7Fe–9S–Mo–C]-homocitrate, and provides a catalytic site for N_2_ reduction. Many of the genes associated with N_2_ fixation are designated as *nif* genes.

Previous studies suggested the evolutionary emergence of N_2_-fixing organisms, termed diazotrophs, from anaerobic archaea (Mus *et al.*, 2019; Pi *et al.*, 2022). The capacity for N_2_ fixation was so advantageous for habitat expansion that it spread over diverse phylogenetic groups of archaea and bacteria and eventually to obligate aerobes. However, all known nitrogenases are structurally related and are intrinsically sensitive to oxygen (O_2_) damage in their associated metalloclusters. Therefore, diazotrophs had to develop various strategies to cope with O_2_ damage under the selective pressure of increasing O_2_ concentrations in the biosphere. One of these strategies was to reduce intracellular O_2_ concentrations even under ambient conditions. This strategy was detected in *Azotobacter vinelandii*, an obligately aerobic and heterotrophic γ-proteobacterium used as a model in N_2_ fixation studies, and includes a process called respiratory protection (Jones *et al.*, 1973; Martin del Campo *et al.*, 2022). This bacterium possesses a complex respiratory electron transport chain consisting of several branches; following an increase in the concentration of O_2_, the branch coupling only partially with proton translocation is specifically up-regulated, thereby increasing O_2_ consumption before its penetration of the cell (Wu *et al.*, 1997). Another strategy is the strict regulation of nitrogenase synthesis at the transcriptional level in response to surrounding O_2_ concentrations as well as the availability of fixed nitrogen and carbon sources. In many proteobacteria, the transcription of *nif* genes is driven by the transcriptional regulator NifA coupled with the RNA polymerase sigma factor σ^54^, and NifA activity is negatively modulated by excess O_2_. In γ- and β-proteobacteria, the NifL protein has been shown to form an inhibitory complex with NifA in response to O_2_ (Martinez-Argudo *et al.*, 2004). In α-proteobacteria, the majority of which lack NifL, NifA activity is considered to be intrinsically sensitive to O_2_ (Dixon and Kahn, 2004).

Diverse aerobic diazotrophs appear to have developed their own strategies to specific physiological and metabolic conditions. Among them, aerobic methane (CH_4_)-oxidizing bacteria, or methanotrophs, are noteworthy. They are obligate aerobes that use CH_4_ as the sole carbon and energy source through the activity of methane monooxygenase (MMO). Two MMOs with different evolutionary origins are present in nature: soluble MMO (sMMO; the *mmo* gene product) in a subset of methanotrophs and intracytoplasmic membrane-bound particulate MMO (pMMO; the *pmo* gene product) in nearly all methanotrophs (Semrau *et al.*, 2010). sMMO and pMMO catalyze the breaking of the strong C–H bond of CH_4_ by using O_2_ within the cell. Methanotrophs are‍ ‍classified into the classes *Alphaproteobacteria* (called type II), *Gammaproteobacteria* (called type I), and *Methylacidiphilae* (the phylum *Verrucomicrobia*) (called type III) (Knief, 2019). The capacity of diazotrophic growth and/or the presence of *nif* genes have been found in virtually all known type II methanotrophs and in the majority of known type I methanotrophs (Murrell and Dalton, 1983; Auman *et al.*, 2001; Dedysh *et al.*, 2004; Hara *et al.*, 2022; Bao *et al.*, 2025). Moreover, the co-occurrence of N_2_ fixation and CH_4_ oxidation was confirmed at the single-cell level for type II methanotrophs using a combination of fluorescence *in situ* hybridization and NanoSIMS (Hara *et al.*, 2022). We inferred a constraint imposed on the protection of nitrogenase in aerobic methanotrophs because the strategy to reduce intracellular O_2_ concentrations may not be compatible with the efficiency of CH_4_ oxidation in the cytoplasm.

In the present study, we aimed to reveal a strategy employed by methanotrophs to cope with O_2_ damage to nitrogenase from the aspect of the regulatory mechanism of *nif* genes. We used *Methylosinus* sp. 3S-1, a type II methanotroph, which was previously shown to exhibit CH_4_ oxidation-dependent activity for N_2_ fixation under 10% (v/v) O_2_ conditions (Shinoda *et al.*, 2019). The results obtained herein showed that the transcription of *nif* genes was markedly enhanced following an increase in the concentration of O_2_. This characteristic regulation may be involved in the strategy employed by *Methylosinus* sp. 3S-1 to maintain N_2_ fixation on the background of aerobic CH_4_ oxidation.

## Materials and Methods

### Genome anal­ysis

The genome DNA library of *Methylosinus* sp. 3S-1 was prepared with SMRTbell Express Template Prep Kit 2.0 (Pacific Biosciences of California) and with a cut-off at 20–25‍ ‍kb using the BluePippin size selection system (Sage Science). The library was sequenced with the PacBio Sequel II system (Pacific Biosciences of California). Reads were assembled using SMRTlink v10.2. The circularity of contigs was confirmed by Circulator v1.5.5 (Hunt *et al.*, 2015). Sequences were annotated using the DFAST web service (Tanizawa *et al.*, 2018). Harr plots were created with GenomeMatcher (Ohtsubo *et al.*, 2008). Average nucleotide identity (ANI) was calculated on the JSpeciesWS web server (Richter *et al.*, 2016).

### Bacterial growth conditions

*Methylosinus* sp. 3S-1 was grown in liquid nitrate mineral salt (NMS) medium (Whittenbury *et al.*, 1970), in which the concentration of KNO_3_ was modified to 0.05% (w/v) (hereafter N-containing medium), in a bottle sealed with a butyl rubber stopper. CH_4_ was added into the bottle with a syringe to 20% (v/v) of the headspace, unless otherwise specified. The culture bottle was incubated at 30°C with shaking. When necessary, the atmosphere in the headspace was initially replaced with N_2_ at the appropriate tension using a vacuum line equipped with a pressure gauge (AVG-300; Okano Works) and other gases were then added with a syringe to the bottle at the necessary volume. The volume ratio of the medium to the headspace in the bottle was set to 1:10, and the initial concentrations of O_2_, CH_4_, and CO_2_ were set to 2–20% (v/v), 5% (v/v), and 0.5% (v/v), respectively, with N_2_ as the balance gas. To achieve diazotrophic growth, KNO_3_ was excluded from N-containing medium (hereafter N-free medium). To deprive the culture medium of nitrogen sources, bacterial cells grown to an optical density at 600‍ ‍nm (OD_600_) of 0.3–0.5 were washed with saline (10‍ ‍mM KCl and 4‍ ‍mM MgSO_4_) by centrifugation, suspended in N-free medium to an OD_600_ of 0.05, and placed in a bottle to be sealed. To achieve different O_2_ concentrations during diazotrophic growth, bacterial cells grown in N-free medium to an OD_600_ of 0.15–0.22 were diluted with N-free medium to an OD_600_ of 0.05. To evaluate N_2_-fixing activity, the atmosphere within a 1,150-mL bottle containing 100‍ ‍mL of the culture sample was initially replaced with argon gas and then with ^15^N-N_2_ (98 atom%; Shoko Science) at a tension corresponding to 74.5% (v/v) (in the case of the 20% O_2_ condition) or 92.5% (v/v) (in the case of the 2% O_2_ condition) of the headspace. O_2_, CH_4_, and CO_2_ were added with the syringe at the necessary volumes.

### Treatment of culture samples

The OD_600_ of culture samples was measured using a UV-1700 spectrophotometer (Shimadzu). Cell density was assessed by a microscopic count with a hemocytometer. In the RNA anal­ysis, culture samples were quickly mixed with a phenol/ethanol solution to yield 1% (v/v) phenol, chilled on ice, and centrifuged. Pelleted cells were suspended in TRIzol Reagent (Invitrogen) and lysed by‍ ‍the FastPrep homogenizer with Lysing Matrix B (MP Biomedicals). Total RNA was prepared according to the instructions of TRIzol and purified further with DNase I (Qiagen) and RNA Clean & Concentrator-25 (Zymo Research) according to the manufacturers’ instructions (the protocol for the purification of >200 nt RNAs was used in the latter kit). RNA yield was measured with an Agilent 2200 TapeStation System (Agilent Technologies). In the protein anal­ysis, culture samples were quickly mixed with trichloroacetic acid to 10% (w/v), chilled on ice, and centrifuged. Pelleted cells were washed with acetone, dried, suspended in a solution (25‍ ‍mM TrisHCl, 0.1‍ ‍mM EDTA, 40‍ ‍mM NaCl, 0.5% Triton X-100, and 0.5% Tween-20, pH 7.5), and sonicated with a Bioruptor (Sonicbio). Total protein concentrations were assessed with a DC Protein Assay (Bio-Rad Laboratories). To measure CH_4_ and O_2_ consumption in the culture, the headspace gas was sampled at intervals. CH_4_ was evaluated with the GC-2025 gas chromatograph (Shimadzu) equipped with a SH-Alumina BOND/Na_2_SO_4_ column (Shimadzu) and flame ionization detector using N_2_ as the carrier gas and temperatures of 40, 100, and 130°C for the column, injector, and detector, respectively. O_2_ was measured using a GC-2014 gas chromatograph (Shimadzu) equipped with a Molecular Sieve 5A column and thermal conductivity detector using helium as the carrier gas and temperatures of 60, 100, and 100°C for the column, injector, and detector, respectively.

### Measurement of N_2_ fixation using ^15^N_2_

A culture (70‍ ‍mL) grown in the presence of ^15^N_2_ was chilled on ice and centrifuged. Pelleted cells were washed with cold saline, suspended in water, transferred into a tin foil cup, and dried in a desiccator with P_2_O_5_. The concentration of ^15^N was measured by Shoko Science with the Flash2000-DELTAplus Advantage ConFloIII System (ThermoFisher Scientific).

### Western blot anal­ysis

Cell lysates containing 1‍ ‍μg of total protein were resolved by SDS-polyacrylamide gel electrophoresis and transferred onto an Immobilon-P PVDF membrane (Millipore). To detect NifH, anti-NifH chicken polyclonal IgY (Agrisera) and HRP-conjugated goat anti-chicken IgY (Arigo Biolaboratories) antibodies were used as the primary and secondary antibodies, respectively. To detect RpoH, anti-*Sinorhizobium meliloti* RpoH1 rabbit antiserum (Mitsui and Minamisawa, 2017) and a HRP-conjugated goat anti-rabbit IgG antibody (ProteinTech Group) were used. We detected chemiluminescence emitted from the target protein with Western Lightning ECL Pro (PerkinElmer) as a substrate using the ChemiDoc XRS+ System (Bio-Rad Laboratories).

### Quantitative reverse-transcription PCR (qRT-PCR)

Complementary DNA was synthesized from total RNA using the PrimeScript RT reagent kit with random hexamers (Takara Bio). Real-time PCR was performed using the ten-fold diluted cDNA solution as the template and the primer pairs listed in Table S1 on the CFX Connect Real-Time PCR Detection System (BioRad Laboratories) using KAPA SYBR FAST qPCR Master Mix (2×) Universal (Kapa Biosystems). To select a stably expressed gene during changes in growth conditions, the quantification cycle (Cq) values of candidate genes in the real-time PCR anal­ysis were subjected to calculations with the geNorm2 (Vandesompele *et al.*, 2002) and BestKeeper (Pfaffl *et al.*, 2004) algorithms implemented in the R package ‘ctrlGene.’ Fold changes in gene expression were calculated by the ΔΔCq method after amplification efficiencies were confirmed to range between 95 and 99% for all the primers used.

### Statistical and phylogenetic anal­yses

Statistical anal­yses were performed using the base R functions aov() for an anal­ysis of variance and TukeyHSD() for Tukey’s Honest Significant Difference post-hoc test, along with the dplyr package for data summarization (Wickham *et al.*, 2023). Data visualization was performed using the ‘ggplot2’ package (Wickham, 2016) in the R computing environment (version 4.3.2; R Core Team, 2023). Phylogenetic trees were constructed using Molecular Evolutionary Genetics Analysis software (version 11; Tamura *et al.*, 2021).

### Accession numbers for the genome sequence

The nucleotide sequences of the 3S-1 genome were deposited in DDBJ under accession numbers AP040855 to AP040857.

## Results

### Complete genome sequence of *Methylosinus* sp. 3S-1

A draft genome sequence of *Methylosinus* sp. 3S-1 was previously reported (Bao *et al.*, 2016). To conduct an extensive survey for N_2_ fixation-related genes, we finished the whole genome sequence using the PacBio RS II single-molecule real-time platform. The genome was 4,967,063 bp (65.9‍ ‍mol% DNA G+C content) and consisted of a circular chromosome (4,502,247 bp; 66.0‍ ‍mol% G+C) and two circular plasmids, p3S1-1 (284,541 bp; 66.0‍ ‍mol% G+C) and p3S1-2 (180,275 bp; 63.6‍ ‍mol% G+C). In comparisons with‍ ‍*Methylosinus trichosporium* OB3b, the genome of which consists of a chromosome (4,496,051 bp) and two plasmids (285,905 and 180,306 bp) (accession no. NZ_ADVE02000001.1 to NZ_ADVE02000003.1) (Stein *et al.*, 2010), ANI values were 99.97, 99.98, and 99.99% for the chromosome, p3S1-1, and p3S1-2, respectively, to their counterpart replicons. A Harr plot anal­ysis showed collinearity over the lengths of the respective pairs, except for a 52.8-kb inversion on the chromosome (Fig. S1). This result indicates that 3S-1 is a close relative of *M. trichosporium* OB3b.

### *Methylosinus* sp. 3S-1 is highly tolerant of O_2_ in diazotrophic growth

**Fig. 1.**
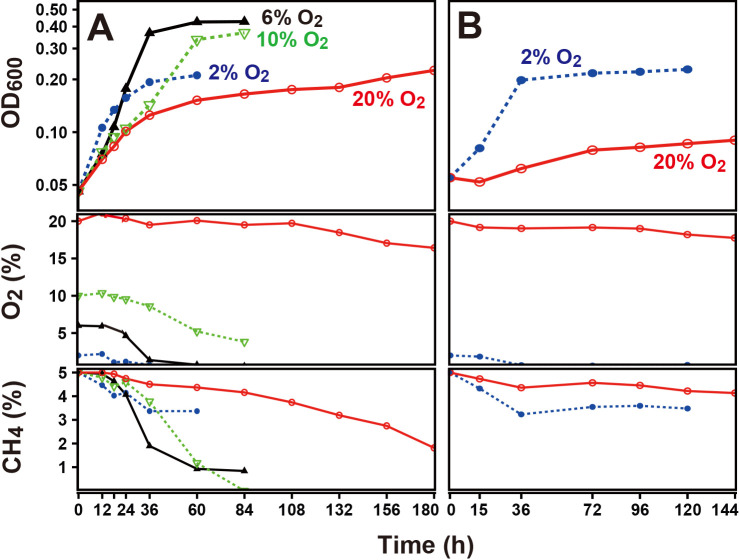
Effects of O_2_ on the growth of *Methylosinus* sp. 3S-1 in N-free medium. Growth was monitored over time using OD_600_ of the medium and O_2_ and CH_4_ concentrations in the headspace. CH_4_ was initially adjusted to 5% (v/v). (A) Cells grown in N-containing medium were rinsed and suspended in N-free medium to an OD_600_ of 0.05. To begin the culture, the O_2_ concentration was adjusted to 2, 6, 10, or 20% (v/v). (B) The 20% O_2_ culture with N-free medium was diluted to an OD_600_ of 0.05 for the growth test with 2 or 20% (v/v) O_2_. The same result was obtained from an independent experiment.

We attempted to examine the effects of higher O_2_ concentrations on the growth of *Methylosinus* sp. 3S-1 in N-free medium. The culture was prepared with N-free medium in sealed bottles in which the headspace was set initially to 2, 6, 10, or 20% (v/v) O_2_, in combination with 5% (v/v) CH_4_ and 0.5% CO_2_ (v/v) with N_2_ as the balance gas, and OD_600_ and O_2_ and CH_4_ concentrations were monitored. OD_600_ increased in all cultures regardless of the initial O_2_ concentration; however, the initial rate of the increase was lower under higher O_2_ conditions (Fig. 1A). OD_600_ reached a plateau following O_2_ depletion in the cultures with initial O_2_ concentrations of 2 and 6% and following CH_4_ depletion in the culture with an initial O_2_ concentration of 10%. In the culture with an initial O_2_ concentration of 20%, the slowest, but most steady increase in OD_600_ was observed until 180‍ ‍h with O_2_ remaining at >17% and CH_4_ at >1.8%. In this culture, the increase in OD_600_ accelerated earlier (~36‍ ‍h) and then became steady even after the culture was continued by diluting and resetting the O_2_ concentration to 20% (Fig. 1A and B). When the same diluted culture was reset to 2% O_2_, rapid growth began immediately (Fig. 1B). We also tested 3S-1 growth in N-containing medium. In this case, the strain exhibited similar growth between the cultures with initial O_2_ concentrations of 2 and 20% (v/v) (Fig. S2), confirming that the negative effect of O_2_ on growth was only observed with N-free medium.

To comparatively characterize N_2_ fixation at low and high O_2_ concentrations, we conducted O_2_ shift experiments on 3S-1 from 2% to higher concentrations in N-free medium; the culture was additionally provided with CH_4_ and CO_2_ (initially 5 and 0.5% [v/v], respectively) with N_2_ as the balance gas. We used ^15^N-labeled N_2_ (98 atom%) as the balance gas when the culture grown at 2% O_2_ (OD_600_ of 0.21) was diluted to OD_600_ of 0.05 and the new O_2_ concentration was set to 2 or 20% (v/v). We measured ^15^N concentrations in cells grown under each O_2_ condition. In the culture with an initial O_2_ concentration of 2%, it increased to 72.2 atom% excess at 18‍ ‍h from a natural abundance level, along with increases in OD_600_ and cell density (Table 1). In the culture with an initial O_2_ concentration of 20%, increases in the ^15^N concentration and cell density were negligible at 18‍ ‍h whereas OD_600_ doubled during the same time. In an extension to 96 h, the ^15^N concentration reached 30.5 atom% excess (Table 1). Although it was not possible to specify a range of O_2_ concentrations that sustained diazotrophic growth, these results indicate that 3S-1 was capable of N_2_ fixation across a wide range of O_2_ concentrations. To examine the relationship between O_2_ concentrations and the rates of diazotrophic growth, a continuous culture, such as turbidostat, will be necessary in the future. The transiently accelerated increase in OD_600_ after the 20% O_2_ shift involved neither substantial N_2_ fixation nor a cell number increase, suggesting that cells were producing absorbance-contributing substances against the background of active CH_4_ oxidation before N_2_ fixation occurred.

### Expression of nitrogenase and mRNAs of related genes under high O_2_ conditions

**Fig. 2.**
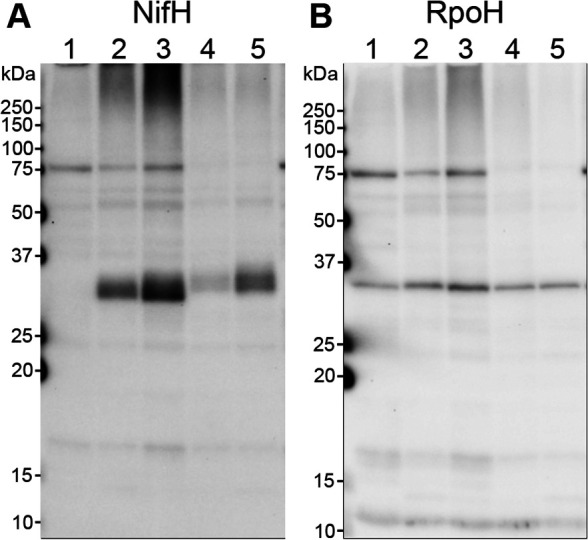
Effects of O_2_ on NifH protein levels in *Methylosinus* sp. 3S-1 cells. A Western blot anal­ysis was conducted using anti-NifH (panel A) and anti-RpoH (panel B) antibodies for cell lysates (0.5‍ ‍μg total protein per lane for the NifH anal­ysis and 1‍ ‍μg total protein per lane for the RpoH anal­ysis). Cells were grown in N-containing medium under an ambient condition supplemented with CH_4_ (5%, [v/v]) (lane 1) or in the same cultures as those in Table 1, where N-free medium was used with 5% (v/v) CH_4_ and the specified initial concentrations of O_2_ (lanes 2–5). Lane 2, cells from the preculture (2% O_2_) for samples in lanes 3–5; lane 3, cells grown for 18‍ ‍h at 2% O_2_ (v/v); lane 4, cells grown for 18‍ ‍h at 20% O_2_ (v/v); lane 5, cells grown for 96‍ ‍h at 20% O_2_ (v/v). The positions of molecular weight markers are indicated on the left side of each panel. The same result was obtained from a set of separate cultures grown in parallel.

We used the same ^15^N-fed culture to investigate whether nitrogenase protein and *nif* mRNA expression was consistent with the N_2_ fixation rate. A Western blot anal­ysis using the anti-NifH antibody detected a single band close to the predicted molecular mass of NifH (31.7‍ ‍kDa) from cells grown in N-free medium with an initial O_2_ concentration of 2% (in both the preculture and the control at 18‍ ‍h), but no corresponding band from cells grown in N-containing medium (Fig. 2A). Eighteen hours after the shift to 20% O_2_, the NifH band showed an upshift in mobility and a decrease in intensity from that at 2% O_2_. By 96 h, the NifH band recovered partially in intensity with the upshifted position (Fig. 2A). In contrast, the band intensity of RpoH, the RNA polymerase sigma factor σ^32^ (33.4‍ ‍kDa), remained constant among the same set of samples as that for NifH (Fig. 2B). These results indicate that the change in NifH levels correlated with that in N_2_ fixation rates. The band shift at high O_2_ concentrations was not unprecedented because NifH of *M. trichosporium* OB3b divided into two distinct bands, the upper of which became solely detected at a high O_2_ concentration in the mutant possessing constitutively expressed sMMO and increased O_2_ tolerance in N_2_ fixation (Kim and Graham, 2001). In the same study, the NifH band disappeared from the wild type at a high O_2_ concentration, which may correspond to 3S-1 where NifH levels initially decreased after the 20% O_2_ shift. Since 3S-1 and OB3b each harbor a single copy of *nifH* in their genomes, the differentiation of NifH by electrophoretic mobility was attributed to post-translational modifications.

Regarding the mRNA levels of *nif* genes, we attempted to identify a suitable reference gene to normalize 3S-1 mRNA data from various growth conditions. We evaluated the stability in expression of eight housekeeping genes (*clpX*, *dnaK*, *gyrB*, *recA*, *rho*, *rpmH*, *rpoB*, and *rpoD*) in cells growing on CH_4_ in (i) N-containing medium at an ambient O_2_ concentration, (ii) N-free medium at 2% O_2_, and (iii) N-free medium at 20% O_2_ at three culture time points (for details, refer to the legend of Fig. S3). We selected *recA* as the reference because its expression was judged to be the most stable under the conditions tested (Fig. S3 and Table S2).

In the 3S-1 genome, many nitrogenase-related genes clustered into putative operons, each of which was preceded by a possible NifA-binding motif and σ^54^-recognized “–24/–12”-type promoter sequences (Fig. S4). This finding strongly suggests that the transcription of these genes is regulated by NifA, as is the case with many proteobacterial diazotrophs (Dixon and Kahn, 2004). We subjected *nifH*, *nifB*, the leading genes of the putative operons, and *nifA* to a qRT-PCR anal­ysis with the O_2_ shift from 2 to 20% (v/v) in N-free medium. The results obtained showed that the mRNA levels of these *nif* genes markedly increased upon the O_2_ shift, in contrast to their protein levels and N_2_ fixation rates (Table 2). Therefore, O_2_ appeared to enhance, not repress, the transcription of the *nif* genes in 3S-1, which is opposite to its effects in many obligate aerobes and facultative anaerobes (Dixon and Kahn, 2004). High mRNA levels were maintained at 96 h, parallel with steady growth based on a low rate of N_2_ fixation (Table 2).

### Effects of O_2_ concentrations and growth states on the expression of *nif* genes

**Fig. 3.**
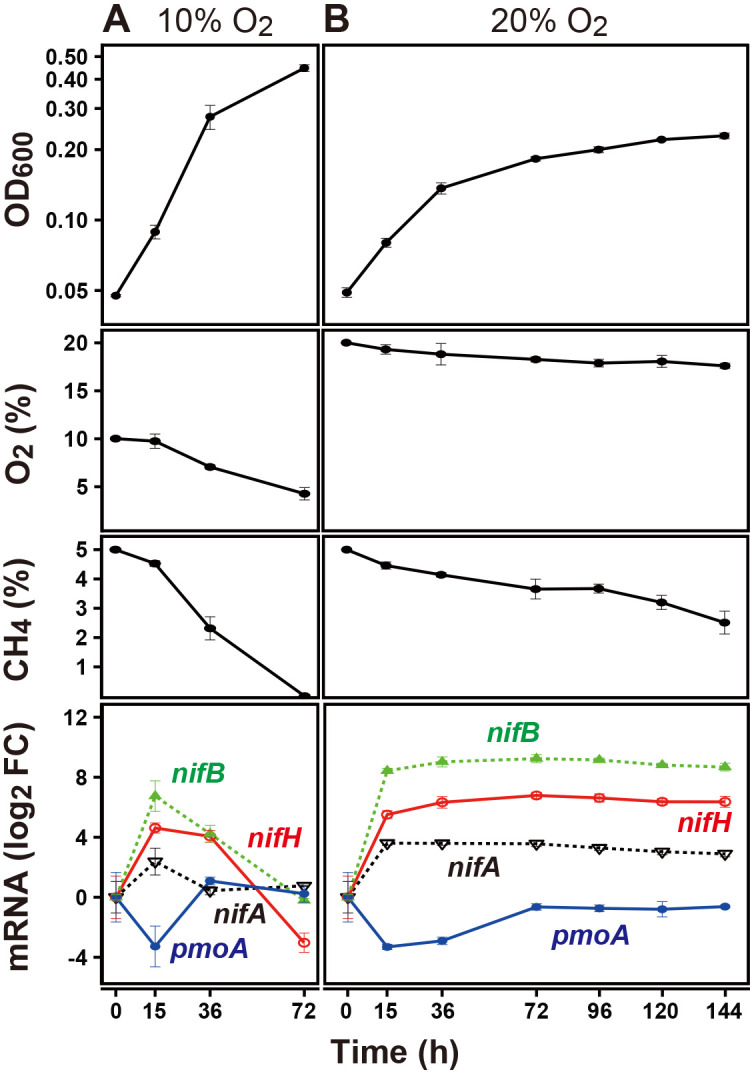
Time-course changes in mRNA levels of *nif* genes in *Methylosinus* sp. 3S-1 during growth in N-free medium following an increase in the O_2_ concentration. A 3S-1 preculture grown in N-free medium at 2% (v/v) O_2_ (Time 0 in the graph) was diluted to an OD_600_ of 0.05 and the culture was initiated at an O_2_ concentration 10% (v/v) (panel A) or 20% (v/v) (panel B). Culture fluid and headspace gas were taken at intervals to assess the OD_600_, mRNA levels of *nifH*, *nifB*, *nifA*, and *pmoA*, and O_2_ and CH_4_ concentrations. Values are the means±SD of four biologically independent measurements and error bars indicate SDs.

**Fig. 4.**
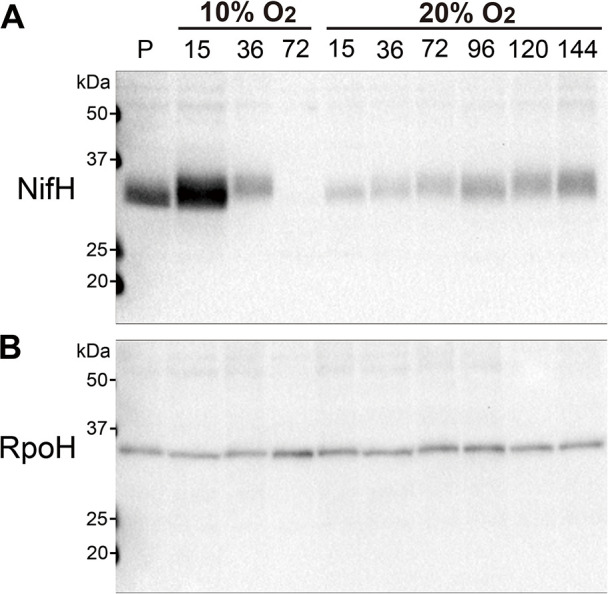
Time-course changes in NifH protein levels in *Methylosinus* sp. 3S-1 during growth in N-free medium following an increase in the O_2_ concentration. Cell lysate samples were taken from the same culture as that in Fig. 3 and subjected to a Western blot anal­ysis using anti-NifH (panel A) and anti-RpoH (panel B) antibodies (a lysate containing 1‍ ‍μg total protein was applied to each lane in both cases). Time (h) after an O_2_ shift to 10 or 20% (v/v) is indicated above each lane; lane P indicates the preculture, which was grown at 2% (v/v) O_2_. The positions of molecular weight markers are indicated on the left side of each panel.

To focus on changes during diazotrophic growth under different O_2_ conditions, we monitored 3S-1 growth in N-free medium over time to compare the expression of *nif* genes and *pmoA*. Following the O_2_ shift from 2 to 10% (v/v), the mRNA levels of *nifH*, *nifB*, and *nifA* were 18-, 96-, and 6-fold higher, respectively, after 15‍ ‍h than those under 2% O_2_ culture conditions. The mRNA levels of these genes then returned to the 2% O_2_ culture levels or lower by 72‍ ‍h while O_2_ and CH_4_ levels were decreasing (Fig. 3A). On the other hand, the NifH protein was detected at 15‍ ‍h as a more intense and larger band upward of that from the 2% O_2_ culture. Only the upper portion of the band remained with decreased intensity at 36 h, and it disappeared below the detection limit by 72‍ ‍h (Fig. 4A). Following the shift from 2 to 20% O_2_ (v/v), the mRNA levels of *nif* genes markedly increased at 15 h, as described above, and were 26- and 364-fold higher than 2% O_2_ culture levels for *nifH* and *nifB*, respectively (Fig. 3B). These high levels continued up to 144‍ ‍h while O_2_ remained at >17% and CH_4_ at >2.5% (Fig. 3B). NifH protein levels decreased at 15‍ ‍h and then gradually increased with an upshift in the band position (Fig. 4A). In contrast to the *nif* genes, the mRNA level of *pmoA* decreased at 15‍ ‍h under 10 and 20% O_2_; it then recovered to the 2% O_2_ culture level in the 10% O_2_ culture, but only partially in the 20% O_2_ culture (Fig. 3A and B). As a control in the protein anal­ysis, RpoH levels remained unchanged in both the 10% and 20% O_2_ experiments (Fig. 4B). Therefore, the changes observed in mRNA and protein levels were specific to *nif* genes and the NifH protein, respectively. These results indicate that O_2_ concentrations negatively correlated with nitrogenase protein levels and positively correlated with *nif* mRNA levels.

**Fig. 5.**
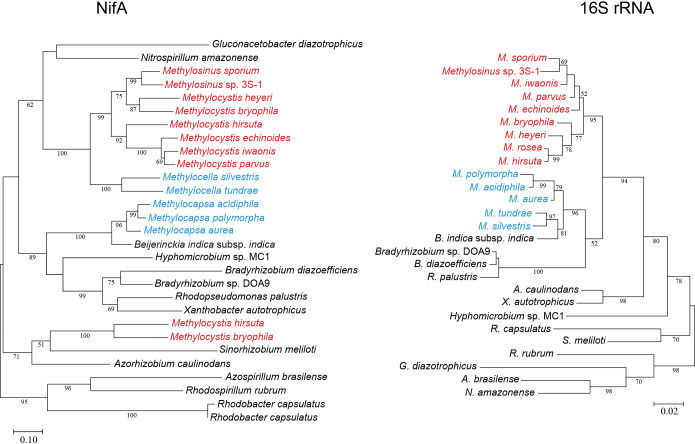
Phylogenetic relationship between α-proteobacterial diazotrophs. Phylogenies based on the amino acid sequences of NifA (left) and nucleotide sequences of 16S rRNA (right) were analyzed using the maximum likelihood method. Species names in red and cyan indicate methanotrophs belonging to the families *Methylocystaceae* (genera *Methylosinus* and *Methylocystis*) and *Beijerinckiaceae* (genera *Methylocella* and *Methylocapsa*), respectively. Note that *Methylocystis hirsuta* and *M. bryophila* each possess two *nifA* homologs. Numerals above branches are the related bootstrap values (%; values ≥50 are shown). Scale bars indicate the substitution number per site.

The transcriptional pattern of *nif* genes was attributed to NifA properties. 3S-1 and other type II (α-proteobacterial) methanotrophs belonging to the genera *Methylosinus*, *Methylocystis*, and *Methylocella* form a distinct clade in the NifA phylogeny, the topology of which differs from that of the 16S rRNA phylogeny (Fig. 5). NifA generally has a domain structure consisting of an N-terminal GAF (cGMP-specific phosphodiesterases, *Anabaena* adenylate cyclases, and *Escherichia coli* FhlA) domain, a central σ^54^-interacting/AAA+ domain, and a C-terminal DNA-binding domain. α-Proteobacterial NifA is further characterized by conserved cysteine residues that are located near the end of the σ^54^-interacting domain and within a linker between the σ^54^-interacting domain and DNA-binding domain. These cysteine residues have been suggested to correlate with intrinsic O_2_ sensitivity (Dixon and Kahn, 2004). Although these cysteine residues are still conserved, *Methylocystaceae* NifAs vary from other NifAs mainly in the GAF domain and interdomain linker (Fig. S5), which may confer unique properties to methanotrophs. Moreover, the increase in *nifA* mRNA levels following the O_2_ upshift may have contributed to the enhanced transcription of other *nif* genes. In 3S-1, the autoactivation of *nifA* expression appeared to occur through a putative NifA/σ^54^-type promoter located across the upstream gene of *nifA* (Fig. S4).

## Discussion

**Fig. 6.**
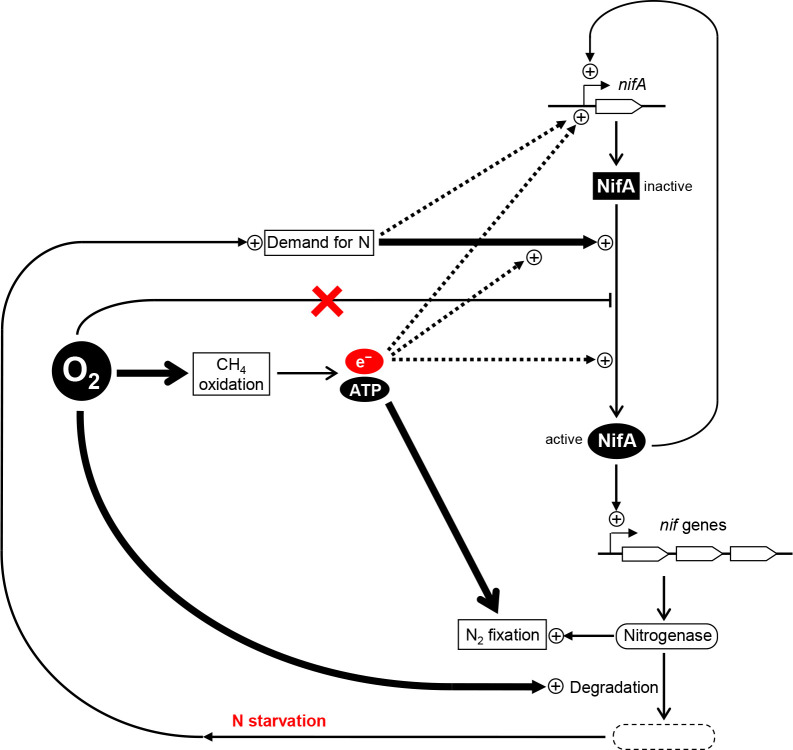
A proposed process in *Methylosinus* sp. 3S-1 to achieve mRNA levels of *nif* genes under high O_2_ conditions. The transcriptional regulator NifA is activated by nitrogen (N) depletion as in many proteobacteria. O_2_ builds up the signal of demand for fixed nitrogen by damaging nitrogenase under diazotrophic conditions. In the case where O_2_ does not inactivate NifA, as in other proteobacteria, the signal may be transduced to directly enhance *nif* transcription under conditions that are unfavorable for the maintenance of nitrogenase activity. Moreover, excess reducing equivalents (e^–^) due to a reduction in nitrogen fixation may serve to promote NifA activity. The autoactivation of *nifA* transcription also contributes to the increase in total NifA activity.

The present study revealed a relationship between the surrounding O_2_ concentration and *nif* mRNA levels in *Methylosinus* sp. 3S-1 during diazotrophic growth. The transcription of *nif* genes in several obligate aerobes and facultative anaerobes is down-regulated as the concentration of O_2_ increases, which is consistent with the intrinsic sensitivity of nitrogenase to O_2_ (Dixon and Kahn, 2004; Martin del Campo *et al.*, 2022). However, *nif* mRNA levels in 3S-1 in the present study were markedly higher at O_2_ concentrations of 10% and 20% (v/v) than 2% (v/v) (Table 2 and Fig. 3), whereas 2% O_2_ appeared to be more favorable for diazotrophic growth than the higher concentrations (Fig. 1). In consideration of the sequence motifs located upstream of the putative *nif* operons, the transcription of *nif* genes may be regulated by NifA and this regulator acts on *nif* promoters in an O_2_-insensitive manner through its intrinsic characteristics or the involvement of an additional protective factor. This notion is consistent with the distinctiveness of methanotrophs in terms of the NifA phylogeny of α-proteobacteria (Fig. 5). In this context, we considered two explanations for the cellular mechanisms underlying the up-regulated transcription of *nif* genes under increased O_2_ conditions. Specifically, O_2_ may promote NifA activity directly or through an unknown regulator that senses O_2_. Alternatively, nitrogenase damage from O_2_ may aggravate the starvation of fixed nitrogen and the imbalance in the cellular redox state, each of which could increase NifA activity (Fig. 6). The present results showed that N_2_ fixation decreased in 3S-1 as the concentration of O_2_ was increased from 2% (v/v) (Fig. 1 and Table 1). On the other hand, CH_4_ oxidation consistently functioned at both 2 and 20% (v/v) O_2_ (Fig. S2) to generate energy and reducing power to be utilized in cellular metabolism. In some α-proteobacteria, such as *Rhodobacter capsulatus* and *Bradyrhizobium diazoefficiens*, excess reducing equivalents contribute to the up-regulated expression of *nif* genes through the RegB/RegA (or homologous RegS/RegR) signal transduction pathway, which is plausible because N_2_ fixation functions as a major sink of electrons (Joshi and Tabita, 1996; Elsen *et al.*, 2000, 2004; Emmerich *et al.*, 2000). We consider it likely that the condition assumed in the second explanation continues as long as 3S-1 is viable for CH_4_ oxidation with nitrogenase activity impaired by O_2_. In any case, enhanced *nif* transcription may increase the synthesis of nitrogenase, which partly compensates for the loss caused by O_2_ damage. The unique pattern of *nif* expression in response to O_2_ may function as a strategy by this methanotroph to maintain N_2_-fixing activity on the background of aerobic CH_4_ oxidation within the same cell.

The present results establish the potential of 3S-1 for N_2_ fixation across a wide range of O_2_ concentrations. We suggested a difference between N_2_ fixation settings under high and low O_2_ conditions. NifH protein levels were lower at an initial O_2_ concentration of 20% than 2% despite the markedly up-regulated transcription (Fig. 2 and 4). The lower protein level is consistent with the low N_2_ fixation rate under the same condition. Moreover, we found that NifH was modified under 10% and 20% (v/v) O_2_ conditions, resulting in changes in its electrophoretic mobility. This NifH modification was also reported in *M. trichosporium* OB3b (Kim and Graham, 2001) and has yet to be characterized. It is important to note that there was a time lag before N_2_ fixation resumed at 20% O_2_ upon a shift from 2% O_2_; OD_600_ markedly increased, whereas cell growth stopped with no N_2_ fixation for this period (Table 1). This implies that the cellular and molecular settings for N_2_ fixation need to be remodeled to adapt to high O_2_ conditions. To elucidate the mechanisms enabling N_2_ fixation under high O_2_ conditions, the molecular events occurring during the time lag need to be exami­ned. Unlike the shift to 20%, 3S-1 cells showed increases in both *nif* mRNA and NifH protein levels following the O_2_ shift from 2 to 10% (v/v) (Fig. 3 and 4). Since diazotrophic growth was slower at 10% O_2_ than at 2% O_2_ (Fig. 1), a fraction of nitrogenase may be inactive, thereby increasing NifA activity through the aforementioned process. Therefore, setting stepwise increases in the concentration of O_2_ in a turbidostat may be useful for investigating the mechanisms by which O_2_ affects each of the ordered steps in nitrogenase turnover, such as the transcription of *nif* genes, the maturation of nitrogenase complexes, inactivation due to metallocluster degradation, and proteolysis.

## Citation

Abdela, A. A., Shinjo, R., Watanabe, T., Asakawa, S., Masuda, S., Shibata, A., et al. (2025) Transcription of Nitrogen Fixation Genes Is Enhanced at Unfavorably High Oxygen Concentrations for Diazotrophic Growth in a Methane-oxidizing Bacterium. *Microbes Environ ***40**: ME25032.

https://doi.org/10.1264/jsme2.ME25032

## Supplementary Material

Supplementary Material

## Figures and Tables

**Table 1.** Incorporation of ^15^N from ^15^N_2_ gas into *Methylosinus* sp. 3S-1 cells during growth in N-free medium^a^

Initial O_2_ conc	OD_600_ and cell density^b^				^15^N conc in cells (atom% excess)^c^		
	0 h	18 h	96 h		0 h	18 h	96 h
2%	0.05 (ND)	0.17 (1.8×10^7^)	ND (ND)		0.0 c	72.2 a	ND
20%		0.10 (4.7×10^6^)	0.17 (1.1×10^7^)			1.4 c	30.5 b

^a^ Samples for ^15^N measurements were taken from a preculture grown in N-free medium at 2% (v/v) O_2_ (0‍ ‍h) and from a culture grown for 18 or 96‍ ‍h after the dilution of the preculture to an OD_600_ of 0.05 and the adjustment of the O_2_ concentration to 2 or 20% (v/v) in the ^15^N_2_ balance; values are means from three separate cultures; ND, not determined. ^b^ Cell density is shown in parentheses as a microscopic cell count mL^–1^. ^c^ Based on 0.366 atom% of natural ^15^N abundance; a significant difference based on Tukey’s test (*P*<0.05) is presented by a different letter following a value.

**Table 2.** Changes in mRNA levels of *nif* genes in *Methylosinus* sp. 3S-1 cells grown at different O_2_ concentrations^a^

Gene	2% O_2_^b^		20% O_2_^b^	
	18 h		18 h	96 h
*nifH*	0.2±0.9		7.9±1.3	8.8±0.5
*nifB*	0.9±0.3		9.0±1.2	9.6±0.4
*nifA*	0.2±1.7		3.0±0.6	2.8±0.1

^a^ Cultures used for the quantitative reverse transcription PCR anal­ysis are the same as those in Table 1. ^b^ Values are the means±SD of the log_2_ fold change in mRNA levels relative to those in the preculture (2% O_2_) from three separate cultures.
