# Supplementary material for: Transcription of Nitrogen Fixation Genes Is Enhanced at Unfavorably High Oxygen Concentrations for Diazotrophic Growth in a Methane-oxidizing Bacterium
Source: Microbes Environ. 2025 Nov 6;40(4):ME25032. doi: 10.1264/jsme2.ME25032 (PMC12727197; doi:10.1264/jsme2.ME25032)
Supplement: Supplementary file 1 — Supplementary Material [file 40_25032_s1.pdf]

Table S1 Primer pairs used for quantitative reverse-transcription PCR

Gene	Sequences (5′→3′)	Amplicon size (bp)
nifA	CGCCGATCTCTATTATCGCATCAG<br>AAAACGTCAGATGAACATTCTGCTC	137
nifH	CGTCATCACCTCGATCAACTTCC<br>CGATGTAGATTTCCTGGGCCTT	143
nifB	GAAGTCACCTATGATCTCGAGTCTC<br>GATCAGGATCTTGACGTCCTCATT	138
pmoA	CACGTCGATGCCGGAATATATCC<br>GAAGTAGACCATCATCGACACGAAG	118
clpX	CAAGGTGCTGGACGATTATGTGATC<br>CGATGAGCAGAATGTTGGATTTGG	143
dnaK	GGTCTCGTTCCTTATAAGATCGTCA<br>CTCCTTCATCTTCTGCAGGATGAA	117
gyrB	CTGATCCTGACCTTCTTCTATCGG<br>GTCCTTGAGATATTGCGTCGATTTG	120
recA	CGTTGAAATTCTACGCTTCGGTG<br>GACCTTGTTCTTGACCACTTTGAC	113
rho	CATCCTCAAGAAGATGTATGTGCTG<br>CAGGTGTTCATCGAATCGAAGAAAG	135
rpmH	GAAGAGAACCTATCAACCCAGCAAG<br>GATAGGGGAGATCAGGCCGAGAG	144
rpoB	GTCAACAAATATGGCTTCATCGAGG<br>GACGTGATATTTCTGCTCTTCCATC	108
rpoD	ATCTCGATCGCCAAGAAATATACGA<br>GGTGGCGTAGGTCGAGAATTTATAG	135

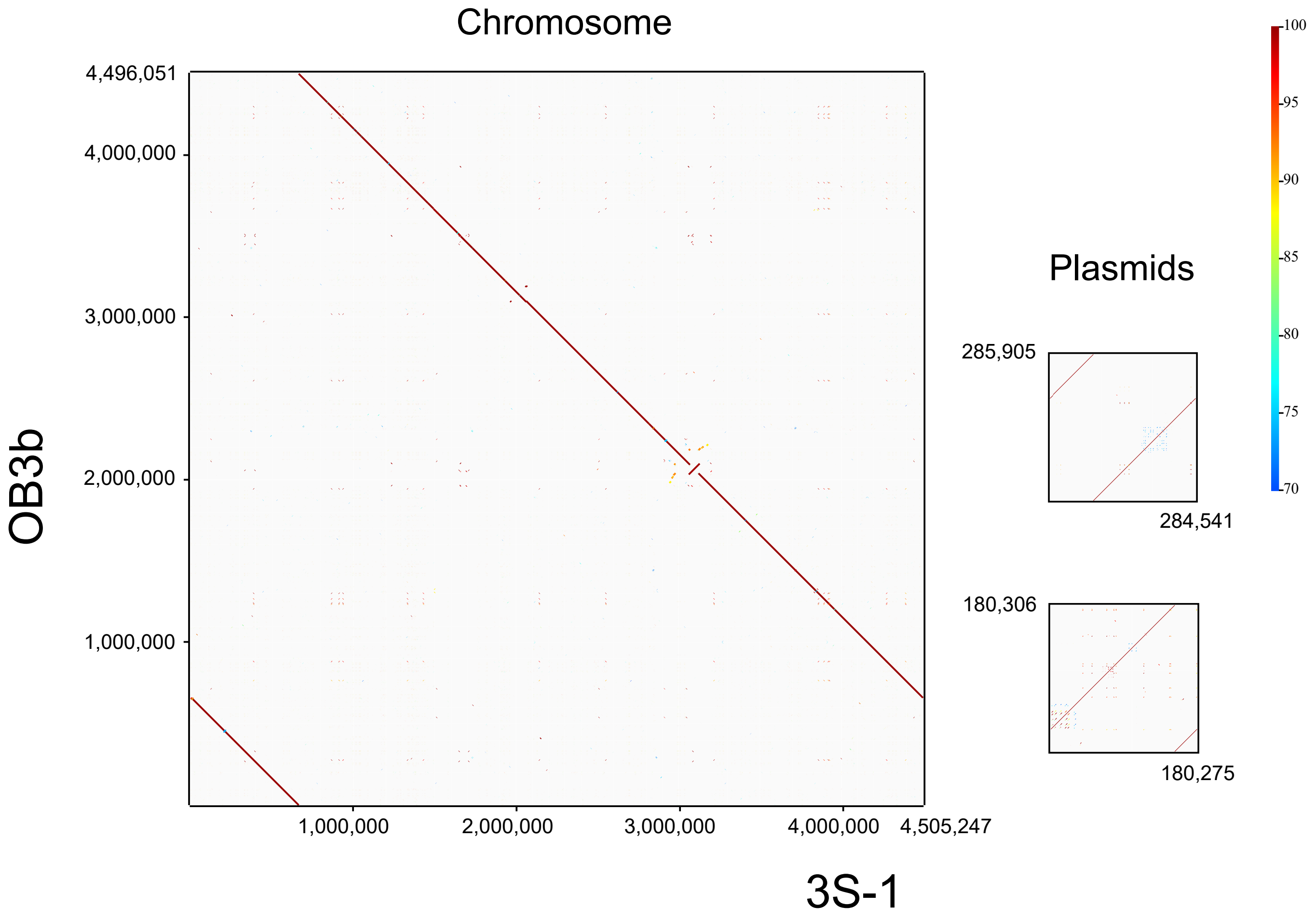

Fig. S1 Harr plot analysis of the genome sequences of *Methylosinus* sp. 3S-1 and *Methylosinus trichosporium* OB3b. The similarity in nucleotide sequences between the counterpart replicons from 3S-1 (horizontal axis) and OB3b (vertical axis) is represented by a color code, whose scale in % identity is shown on the right.

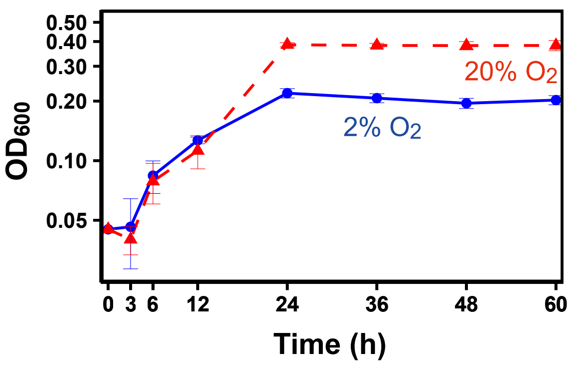

Fig. S2 Growth curves of *Methylosinus* sp. 3S-1 in N-containing medium at different initial O_2 concentrations. The value shown in the graph are mean±SD from three independent cultures at each concentration.

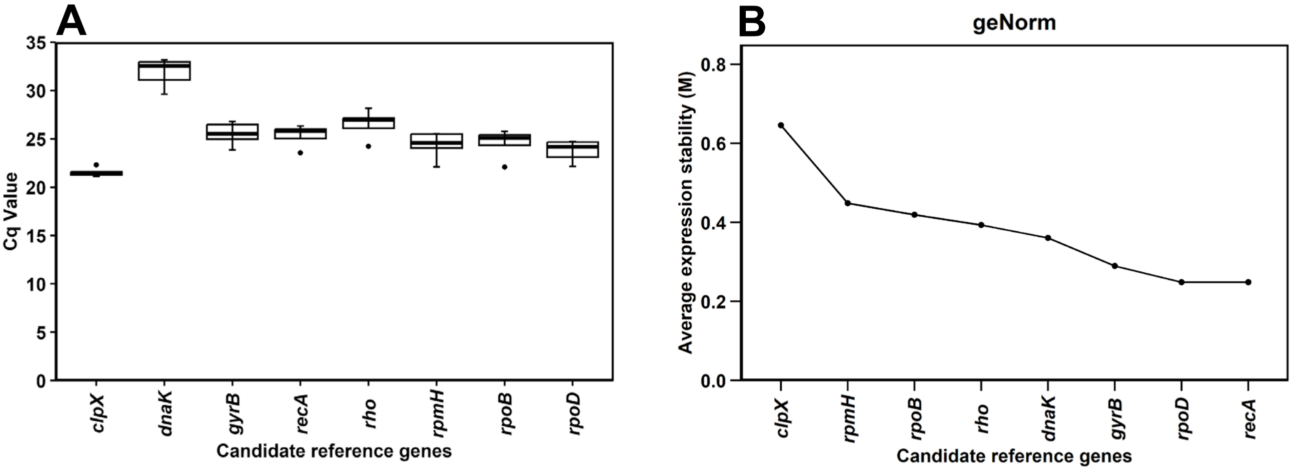

Fig. S3 Expression stability analysis. (A) Quantification cycle (Cq) values of candidate reference genes. Real-time reverse-transcription qPCR output Cq values of eight housekeeping genes in 3S-1 cells growing on CH_4 in (i) N-containing medium at ambient O_2 concentration (at an OD of 0.32), (ii) N-free medium at 2% O_2 (at an OD of 0.14), and (iii) N-free medium at 20% O_2 at three points of culturing time (at ODs of 0.15, 0.17, and 0.25). (B) M-values calculated by geNorm algorithm. These values represent the average pairwise variation between a gene and the other genes, with a lower value indicating greater stability. Here, *recA* and *rpoD* showed the lowest value of 0.249.

Table S2 Expression stability evaluation by BestKeeper algorithm[a]

**Gene name**	***clpx***	***dnaK***	***gyrB***	***recA***	***rho***	***rpmH***	***rpoB***	***rpoD***
Geo Mean [Cq]	21.539	31.857	25.51	25.326	26.492	24.322	24.502	23.749
Min [Cq]	21.113	29.63	23.859	23.554	24.231	22.102	22.081	22.16
Max [Cq]	22.319	33.167	26.799	26.334	28.165	25.526	25.778	24.718
SD [±Cq]	**0.331**	1.212	**0.892**	**0.842**	1.092	1.028	1.065	**0.905**
CV [%Cq]	1.536	3.802	3.493	3.322	4.116	4.223	4.34	3.806
Min [x-fold]	-1.343	-4.683	-3.142	-3.416	-4.794	-4.659	-5.357	-3.007
Max [x-fold]	1.718	2.479	2.444	2.01	3.189	2.304	2.421	1.958
SD [±x-fold]	1.258	2.317	1.855	1.792	2.131	2.04	2.092	1.872
Coeff. of Corr. [r]	0.424	0.989	0.943	**0.997**	0.985	0.973	0.992	0.972
p-value	0.477	0.001	0.016	0	0.002	0.005	0.001	0.006

[Cq] = Cycle quantification; Geo Mean [Cq] = Geometric mean of Cq; Min/Max [Cq] = Lowest and highest Cq values; SD [Cq] = Standard deviation of Cq; CV [%Cq] = Coefficient of variation of Cq (percentage); Min/Max [x-fold] = Lowest and highest expression levels as absolute x-fold regulation values; SD [± x-fold] = Standard deviation of absolute regulation coefficients; Coeff. of Corr. [r] = Correlation coefficient between each candidate and the BestKeeper index.

[a] Relevant outputs are marked in boldface: SD [±Cq] <1 and Pearson's correlation coefficient (r) closest to 1. Here, *recA* is ranked as the most stable gene in these criteria.

A

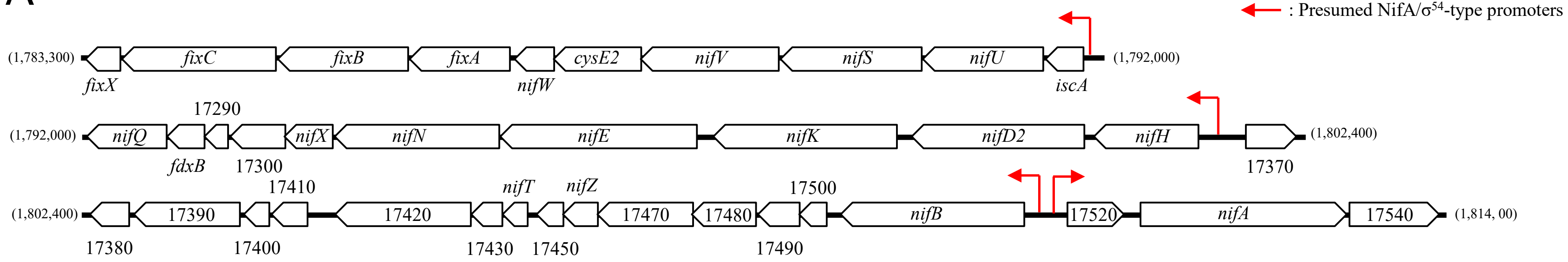

B

```
                NifA-binding                     -24/-12 promoter        Start codon

iscA         CAATGTGGGTTAGTCGACAGCT -(39)- GGTTGGCATAGCGGTTGCTAA -(37)- ATG
nifH         GACTGTCGGCTTTGACACACGT -(65)- GCTTGGCACGCCGCTTGCGAC -(154)- ATG
nifB         CAGTGTAGGCATCTGGACAGAT -(53)- CGATGGCATGCTTGTTGCGAC -(110)- ATG
Ms3S1_17520  TGCTGTCGACAACGCGACACTT -(91)- GATTGGCGCGGCATTTGCCTT -(9)- ATG

Consensus       TGT          ACA                TGGCACG    TTGC
```

Fig. S4 Organization of nitrogen fixation genes on the chromosome of *Methylosinus* sp. 3S-1. (A) A gene map of the region with coordinates from 1,783,300 to 1,814,00 in nucleotide. In the diagram, gene IDs are abbreviated, e.g., “Ms3S1_17290” to “17290”. (B) Comparison of sequences upstream to the leading genes of the putative operons.

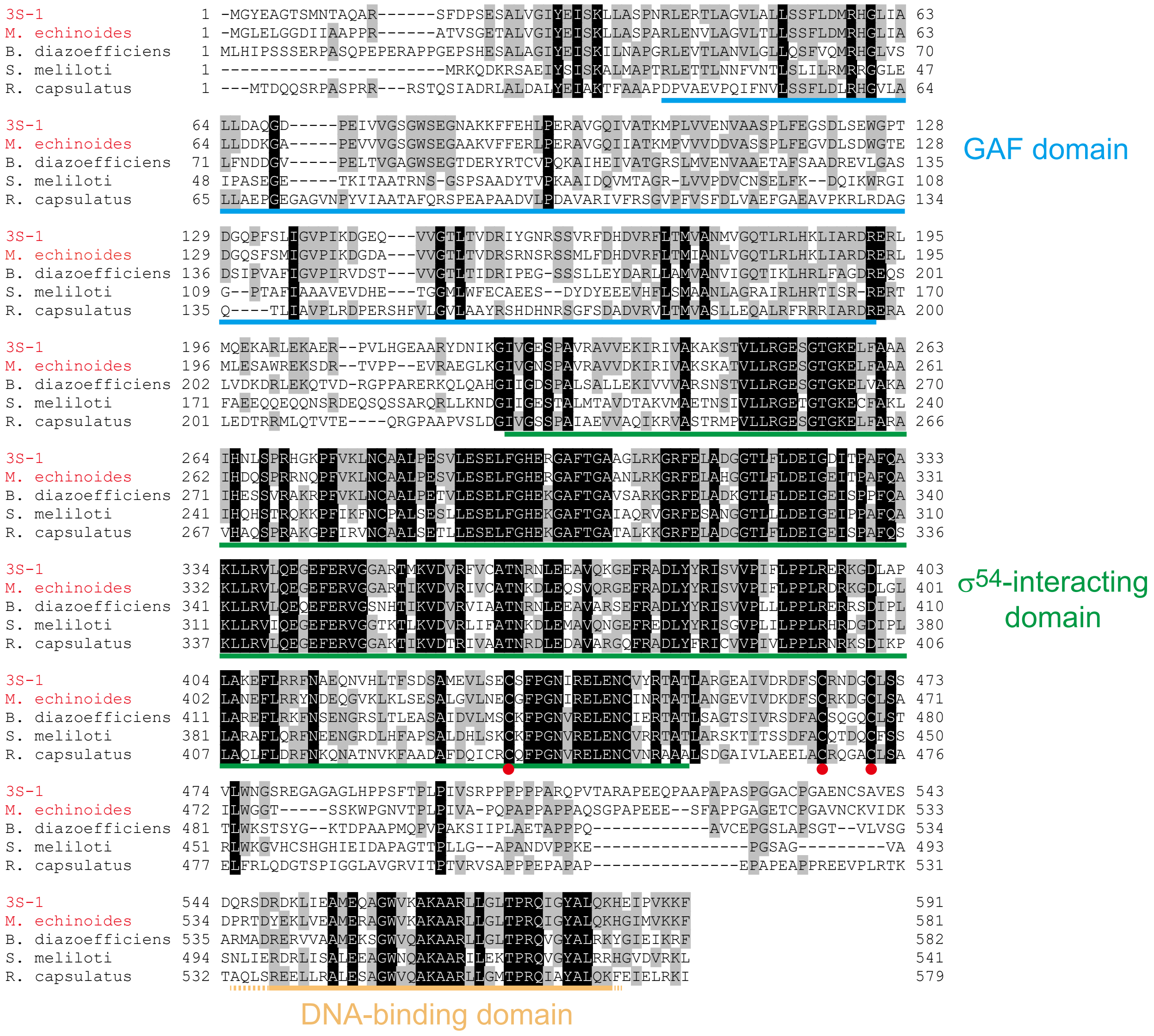

Fig.S5 Comparison of NifA from *Methylosinus* sp. 3S-1, *Methylocystis echinoides*, *Bradyrhizobium diazoefficiens*, *Sinorhizobium meliloti*, and *Rhodobacter capsulatus*. The two methanotroph species are highlighted in red. Amino acid sequences were aligned by using ClustalW. Domain assignment appearing in the UniProt database was used to delimit three domains in the respective sequences. Cysteine residues present exclusively in α-proteobacterial NifA are marked by red circles.
